# Ultrasound enhanced prehospital thrombolysis using microbubbles infusion in patients with acute ST elevation myocardial infarction: *Rationale and design of the Sonolysis study*

**DOI:** 10.1186/1745-6215-9-72

**Published:** 2008-12-10

**Authors:** Jeroen Slikkerveer, Pieter A Dijkmans, Gertjan T Sieswerda, Pieter AFM Doevendans, Arie PJ van Dijk, Freek WA Verheugt, Thomas R Porter, Otto Kamp

**Affiliations:** 1 Department of Cardiology and Institute of Cardiovascular Research, VU University Medical Center, Amsterdam, The Netherlands; 2 University Medical Center Utrecht, Utrecht, The Netherlands; 3 Radboud University Nijmegen Medical Center, Nijmegen, The Netherlands; 4 University of Nebraska Medical Center, Omaha, Nebraska, USA; 5 Interuniversity Cardiology Institute of the Netherlands, Utrecht, The Netherlands

## Abstract

**Background -:**

Experimental studies have shown that ultrasound contrast agents enhance the effectiveness of thrombolytic agents in the presence of ultrasound *in vitro *and *in vivo*. Recently, we have launched a clinical pilot study, called "Sonolysis", to study this effect in patients with ST-elevation myocardial infarction based on proximal lesions of the infarct-related artery.

**Methods/design -:**

In our multicenter, randomized, placebo controlled clinical trial we will include patients between 18 and 80 years of age with their first ST-elevation myocardial infarction based on a proximal lesion of the infarct-related artery. After receiving a single bolus alteplase 50 mg IV (Actilyse^® ^Boehringer Ingelheim GmbH), a loading dose of aspirin 500 mg, and heparin 5000 IU in the ambulance according to the prehospital thrombolysis protocol, patients, following oral informed consent, are randomized to undergo 15 minutes of pulsatile ultrasound with intravenous administration of ultrasound contrast agent or placebo without ultrasound. Afterwards coronary angiography and, if indicated, percutaneous coronary intervention will take place. A total of 60 patients will be enrolled in approximately 1 year.

The primary endpoints are based on the coronary angiogram and consist of TIMI flow, corrected TIMI frame count, and myocardial blush grade. Follow-up includes 12-lead ECG, 2D-echocardiography, cardiac MRI, and enzyme markers to obtain our secondary endpoints, including the infarct size, wall motion abnormalities, and the global left ventricular function.

**Discussion -:**

The Sonolysis study is the first multicenter, randomized, placebo controlled clinical trial investigating the therapeutic application of ultrasound and microbubbles in acute ST-elevation myocardial infarction patients. A positive finding may stimulate further research and technical innovations to implement the treatment in the ambulance and maybe obtain even more patency at an earlier stage.

**Trial registration -:**

Trialregister NTR161

## Background

Thrombo-occlusive cardiovascular disease is the leading cause of mortality in the western world. Among these, acute ST-elevation myocardial infarction (STEMI) is an important disease with high morbidity and mortality. In the course of years, two major treatment strategies have been developed, aiming at immediate restoration of coronary blood flow.

Currently, the first choice of treatment in patients with acute STEMI is primary percutaneous coronary intervention (PCI). However, its feasibility depends on the availability of equipment and trained personnel. As a result, thrombolysis is worldwide the most commonly used therapy for patients with STEMI[1]. Nevertheless, thrombolysis is known to have a relatively low recanalization rate and is related to more haemorrhagic complications[2].

Hence, there is an ongoing search for a non-invasive and easy applicable therapeutic strategy with less serious side-effects, still based on the early restoration of coronary blood flow.

One of the new non-invasive therapy strategies is the use of ultrasound and an ultrasound contrast agent, in combination with thrombolysis[3,4].

Previous studies have indicated that ultrasound alone may accelerate fibrinolysis[5]. The addition of microbubbles being destroyed by high pressure ultrasound leads to an even greater increase of fibrinolysis. There are three proposed mechanisms which lead to this increase. The first is the occurrence of shear stress based on micro-jets which are created after the destruction of the microbubbles by ultrasound[6]. The second mechanism is the generation of reactive oxygen species in endothelial cells[7]. The third proposed mechanism for the increase of fibrinolysis is temperature rise which also takes place after the microbubbles collapse under influence of ultrasound[8]. Although the full mechanism remains to be resolved, the application of ultrasound alone and ultrasound in combination with contrast agents have proven their value by enhancing clot dissolution, by creating microholes in fresh thrombi. Tachibana and Tachibana were among the first to describe the effect of an ultrasound contrast agent on fibrinolysis *in vitro*[9]. They added urokinase with microbubbles (Albunex) and ultrasound, which led to a significant increase in fibrinolysis. This result was confirmed by other *in vitro *studies in the course of years [10-13] (Table 1).

**Table 1:** Studies assessing the influence of microbubbles and ultrasound on thrombolysis

Author(s)/reference	Microbubble	Target *in vivo*/*in vitro*	TL, US, MB	US frequency	Outcome
Tachibana and Tachibana[9]	Albumin MB (albunex)	*In vitro *thrombus	UK, US and MB	170 kHz, 0.5 W/cm^2^	Significantly increased thrombolysis with thrombolytics, US and MB
Kondo et al. [10]	Air-filled MB with galactose/palmitic acid shell	*In vitro *white thrombus	t-PA, US and MB	10 MHz, 0.5–1.0 W/cm^2^	Significant enhancement of thrombus reduction by MB
Nishioka et al. [11]	DDFP	*In vitro *and *in vivo *canine iliofemoral arteries	MB and US	20 kHz, 1.5 W/cm^2^	Increased clot disruption and recanalization with US and MB
Porter et al. [12]	Air-filled MB/PESDA MB	*In vitro *thrombus	UK, US and MB	20 kHz, 40 W/cm^2^	Significant better thrombolysis of PESDA than air-MB. Optimal thrombolysis with UK and MB
Mizushige et al. [13]	Albumin Shell, air-filled/galactose shell air-filled/DDFP-filled MB	*In vitro *thrombus	t-PA, US, MB	10 MHz, 1.02 W/cm^2^	Thrombolysis was greatest in DDFP-MB-group
Birnbaum et al. [14]	PESDA MB	*In vivo *canine iliofemoral artery	US and MB	Up to 160 W/cm^2^	Significant higher recanalization rate with MB
Siegel et al. [15]	DDFP/PESDA	*In vivo *rabit iliofemoral artery and canine LAD	SK, US, MB	20–37 kHz, 1.5–160 W/cm^2^	Increased clot dissolution with US, MB and SK
Culp et al. [16]	PESDA tagged with eptifibatide	*In vivo *pigs ascending pharyngeal artery	US and MB	1 MH, 2 W/cm^2^	Improvement of recanalization rate in eptifibatide tagged PESDA
Xie et al. [17]	Definity	*In vivo *canine femoral artery and vein	US and MB	1 MHz, 0.4–0.6 W/cm^2 ^and 10 W/cm^2^	Higher recanalization rates with MB and US compared to US alone
CLOTBUST investigators[18]	-	*In vivo *middle cerebral artery Phase 2 trial	t-PA and US	2 MHz	Ultrasound augments t-PA induced arterial recanalization
Molina et al. [19]	Galactose MB (Levovist)	*In vivo *middle cerebral artery phase 2 trial	t-PA, US, MB	2 MHz	MB safely induces acceleration of US enhanced thrombolysis
Cohen et al. [20]	-	*In vivo *STEMI phase 2 trial	rt-PA and US	27 kHz	No major adverse events

MB = microbubble; US = ultrasound; TL = thrombolytic; (r)t-PA = (recombinant) tissue plasminogen activator; PESDA = perfluorocarbon-exposed sonicated dextrose albumin; UK = urokinase; DDFP = dodecafluoropentane; LAD = left artery descending; SK = streptokinase

Birnbaum et al. were among the first to conduct an *in vivo *study[14]. They used rabbit iliofemoral arteries to examine the effect of ultrasound and microbubbles on fibrinolysis, without using any fibrinolytics. The recanalization rate in the artery treated with a combination of microbubbles and ultrasound was significantly higher compared to the application of ultrasound or microbubbles alone.

Siegel et al. conducted an other *in vivo *study in rabbit iliofemoral arteries[15]. They showed a significant increase of fibrinolysis in the combination of streptokinase and ultrasound compared to streptokinase alone. Adding microbubbles did not made any difference, however the combination of the three was still better then streptokinase alone.

Other *in vivo *studies confirmed a higher recanalization rate using ultrasound and microbubbles[16,17] (Table 1).

In 2004, the CLOTBUST investigators published results of a multicenter phase II trial conducted in patients with acute stroke[18]. They randomly assigned 126 patients to receive continuous ultrasound or placebo following treatment with intravenous t-PA (0.9 mg/kg with a maximum of 90 mg) within 3 hours after the onset of symptoms. The results within 2 hours after administration showed a significant increase of complete recanalization in the ultrasound group compared to placebo. It also showed a nonsignificant trend towards recovery from stroke, compared with placebo.

Molina et al. conducted a trial in which they added microbubbles on top of ultrasound and t-PA[19]. The addition of microbubbles induced further enhancement of ultrasound augmented thrombolysis, leading to more complete recanalization.

The first to conduct a study in which ultrasound and thrombolytic therapy was combined in a small group of STEMI patients were Cohen et al. They found no major adverse events and regarding efficacy their outcome compared favourably with historical data[20].

However, until now the combination of ultrasound, ultrasound contrast agents (UCA), and thrombolytic therapy has never been tested in STEMI patients. Therefore, we will perform the first study in a pilot design in patients with STEMI, in which we compare the combination of ultrasound and microbubbles with placebo after prehospital thrombolysis.

## Methods and design

### Study design

Sonolysis is designed as a multicenter, randomized, placebo controlled clinical trial that will be conducted in the VU University Medical Center Amsterdam, University Medical Center Utrecht, and Radboud University Nijmegen Medical Center.

We will include patients between 18 and 80 years of age with an acute STEMI based on a proximal lesion. To make sure that we deal with a blocked proximal major epicardial coronary artery our inclusion criteria consist of a sum of ST-elevation of 6 mm or more in combined leads and 1 mm or more ST-elevation in lead V3R when there is an inferior infarct. The exclusion criteria are mentioned in Table 2.

**Table 2:** Exclusion criteria of the Sonolysis study

• Clinical instability	
• Previous Q-wave myocardial infarction	
• Contra indications alteplase (Table 3)	
• Known pulmonary hypertension (> 90 mmHg)	
• Known allergy of Luminity^®^	

Patients are randomized in either a placebo group or a group that receives the ultrasound contrast agent Luminity^®^.

**Figure 1:**
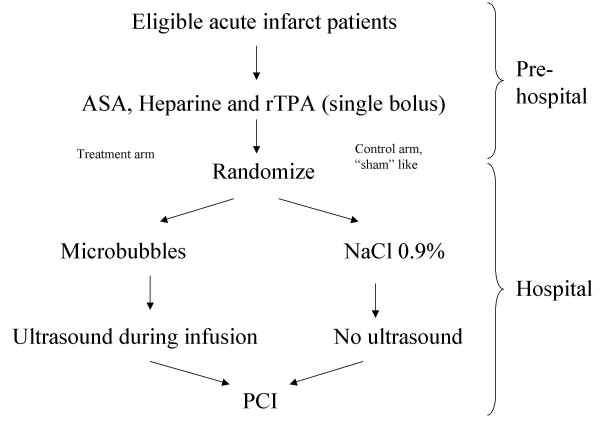
Flow chart of the study design, divided in a hospital and a pre- hospital part.

Randomization will take place on announcement by the ambulance of a patient with an eligible STEMI. This announcement is done through the unique Lifenet system. An ECG of a patient with chest pain is recorded by ambulance personnel and sent to a computer in the coronary care unit of the hospital. There, the cardiology resident on call consults the intervention cardiologist and together they will check the ECG for the inclusion criteria. If the patient is eligible for the study, the ambulance personnel will be informed. This all is summarised in a flow chart (Figure 1)

Randomization will be done by opening a sealed envelope containing the contrast agent Luminity^® ^or saline 0.9%, the placebo.

Patients will be pre-treated in the ambulance with a loading dose of aspirin 500 mg iv., heparin 5000 IU iv., and a single bolus alteplase 50 mg IV (Actilyse^® ^Boehringer Ingelheim GmbH). The latter will be administered in approximately 3 minutes if there are no contra-indications (Table 3). This is in accordance with the prehospital thrombolysis protocol[21,22].

**Table 3:** Contraindications alteplase.

• Reduced consciousness	
• Paralysis	
• Arterial hypertension (systolic pressure > 180 mmHg)	
• Known or suspected haemorrhagic diathesis	
• Possible pregnancy	
• Any history of stroke	
• In the **past 3 months**	
◦ Significant trauma	
◦ Major surgery	
◦ Haemoptysis	
◦ Documented ulcerative gastrointestinal disease	
◦ Manifest or recent severe or dangerous bleeding	
• Allergy to streptokinase or t-PA	
• Systolic blood pressure of less than 80 mmHg during more than 5 minutes with clinical symptoms of shock	

To avoid any time delay, informed consent will be obtained orally, prior to PCI at arrival in the hospital. Written informed consent will be given after primary PCI when the patient is in a stable clinical condition.

At arrival in the hospital, patients receive, after oral informed consent, either placebo (i.e. 50 ml saline 0.9%) without ultrasound, or the ultrasound contrast agent Luminity^® ^during simultaneously pulsatile ultrasound application.

We will use one flacon Luminity^® ^of 1.5 ml which contains 225 microliter perflutren, a high-molecular-weight gas, which increases the stability of the microbubbles. This will be shaken in the Vialmix™ for 45 seconds, to produce the microspheres. After that 48.5 ml of saline 0.9% will be added to create a 50 ml milky white suspension. The suspension or placebo will be administered intravenously in 15 minutes with an infusion rate of 200 ml/h.

Luminity^® ^is registered in the Netherlands as a diagnostic contrast agent to improve imaging quality during echocardiography. Most occurring complications are minor, like headache (2.0%), flushing (1.0%), and back pain (0.9%). Allergic reactions happen approximately in one of every 10.000 patients[23].

The dose of 1.5 ml Luminity^® ^has been shown to be safe and well tolerated. Thus, we expect no difference in adverse events between both groups.

### Endpoints

The primary endpoints of the study are TIMI flow grade, corrected TIMI frame count, and myocardial blush grade, to document the patency of the culprit vessel. All of these are measured during coronary angiography pre-PCI.

Secondary endpoints are ST resolution based on a 12-lead ECG registration during ultrasound, PCI and post-PCI, infarct size derived from measurement of the marker enzymes, and wall motion abnormalities (WMA) measured with 2D-echo and contrast enhanced MRI.

### Cardiac imaging

The ultrasound equipment used in this protocol is the Ie33 (Philips, Eindhoven, the Netherlands). In combination with a X3-1 probe we make 3D full volume images of the aortic root in the parasternal short axis view to ensure that the proximal parts of the epicardial coronary artery system is encompassed within the target zone.

The mechanical index will be set on 1.18 in order to make the microbubble collapse. The frequency we will use is 1.6 MHz intermittently (5 seconds on, 5 seconds off) to ensure the microbubbles to enter the proximal portion of the occluded coronary artery between the pulses.

During follow-up, a 2D echo will be performed to obtain information about wall motion abnormalities, left ventricle volumes, and ejection fraction.

The standard emergency coronary angiogram will be performed to assess the patency of the culprit lesion, the TIMI flow, the corrected TIMI frame count, and the myocardial blush grade. To obtain the myocardial blush grade the angiographic runs will be prolonged until the venous phase of the contrast passage is seen[24]. The frame rate will be set on 12,5 frames/sec. To calculate the corrected TIMI frame count the score will be adjusted for the used frame rate[25]. If necessary, a PCI will be carried out.

Patients are studied on a clinical 1.5 Tesla MRI scanner within 2 to 9 days after primary PCI, and for 4-month follow-up. ECG-gated cine SSFP (Steady State Free Precession) MR images are obtained during repeated breath-holds in the three standard long axis views (four-, three- and two-chamber view). Additional short axis slices are acquired covering the entire left ventricle, to examine regional and global left ventricular function.

After that i.v. injection of Gd-DTPA first-pass perfusion imaging is performed with a saturation-recovery gradient-echo pulse sequence. Delayed contrast-enhanced images are acquired 10 minutes post-contrast with an inversion-recovery gradient-echo pulse sequence to identify the location and extent of myocardial infarction. The data are obtained with slice locations identical to the functional images.

### Follow-up

Follow-up examination will exist from 12-lead ECG registration, 2D-echocardiography, contrast enhanced MRI, and biomarkers of myocardial infarction (CK-MB). All serving to achieve the secondary endpoints of this study.

MRI and 2D-echocardiography will be used to assess the global and regional wall motion, left ventricular function, and left ventricular ejection fraction. 12-lead ECG registration will be used to observe the ST-segment resolution and markers to compare infarct size of both groups.

### Statistical analysis

The aim of this pilot study is to investigate whether the combination of ultrasound and microbubbles enhance clot dissolution after pre-treatment of STEMI patients with a loading dose of aspirin 500 mg, heparin 5000 IU, and alteplase single bolus 50 mg.

The reason we use a single bolus alteplase is to avoid serious adverse events. The PACT-trial[26] showed there was no difference in severe complications between the placebo group and the single bolus alteplase group after pre-treatment with aspirin and heparin, but there still was a significant increase in TIMI 3 flow in the alteplase group (33%) compared to placebo (15%). With our study we are aiming for 65% TIMI 3 flow in the group treated with microbubbles. In order to demonstrate this with a power of 80% and an α of 0.05 (two sided) we need a sample size of 29 patients per group. Therefore, we are intending to randomize 60 patients in this pilot study.

Obtained baseline data will be presented as mean ± standard deviation (SD). Differences between the treatment group, with ultrasound and microbubbles, and the control group in baseline data, biomarkers, 2D-echo, and MRI variables will be assessed by unpaired students t-test.

Chi-square analyses will be conducted to observe differences in primary and secondary endpoints between the treatment group and the control group.

All analysis will be performed based on an intention-to-treat principle.

## List of abbreviations

PCI: percutaneous coronary intervention; SD: standard deviation; SSFP: Steady State Free Precession; STEMI: ST-elevation myocardial infarction; UCA: ultrasound contrast agents; WMA: wall motion abnormalities.

## Competing interests

The authors declare that they have no competing interests.

## Authors' contributions

JS in collaboration with PAD and OK designed the study and drafted the manuscript. GTS, PAFMD, AvD, FWAV and TRP revised the manuscript critically. All have given their final approval of the version to be published.

## References

[B1] Werf F Van de, Ardissino D, Betriu A, Cokkinos DV, Falk E, Fox KA, Julian D, Lengyel M, Neumann FJ, Ruzyllo W, Thygesen C, Underwood SR, Vahanian A, Verheugt FWA, Wijns W (2003). Management of acute myocardial infarction in patients presenting with ST-segment elevation. The Task Force on the Management of Acute Myocardial Infarction of the European Society of Cardiology. Eur Heart J.

[B2] (1993). An international randomized trial comparing four thrombolytic strategies for acute myocardial infarction. The GUSTO investigators. N Engl J Med.

[B3] Dijkmans PA, Juffermans LJ, Musters RJ, van Wamel A, ten Cate FJ, van Gilst W, Visser CA, de Jong N, Kamp O (2004). Microbubbles and ultrasound: from diagnosis to therapy. Eur J Echocardiogr.

[B4] Dijkmans PA (2007). Ultrasound contrast agents: from diagnosis to therapy. PhD Thesis.

[B5] Pfaffenberger S, Devcic-Kuhar B, Kastl SP, Huber K, Maurer G, Wojta J, Gottsauner-Wolf M (2005). Ultrasound thrombolysis. Thromb Haemost.

[B6] van Wamel A, Bouakaz A, Versluis M, de Jong N (2004). Micromanipulation of endothelial cells: ultrasound-microbubble-cell interaction. Ultrasound Med Biol.

[B7] Juffermans LJ, Dijkmans PA, Musters RJ, Visser CA, Kamp O (2006). Transient permeabilization of cell membranes by ultrasound-exposed microbubbles is related to formation of hydrogen peroxide. Am J Physiol Heart Circ Physiol.

[B8] Wu J (1998). Temperature rise generated by ultrasound in the presence of contrast agent. Ultrasound Med Biol.

[B9] Tachibana K, Tachibana S (1995). Albumin microbubble echo-contrast material as an enhancer for ultrasound accelerated thrombolysis. Circulation.

[B10] Kondo I, Mizushige K, Ueda T, Masugata H, Ohmori K, Matsuo H (1999). Histological observations and the process of ultrasound contrast agent enhancement of tissue plasminogen activator thrombolysis with ultrasound exposure. Jpn Circ J.

[B11] Nishioka T, Luo H, Fishbein MC, Cercek B, Forrester JS, Kim CJ, Berglund H, Siegel RJ (1997). Dissolution of thrombotic arterial occlusion by high intensity, low frequency ultrasound and dodecafluoropentane emulsion: an in vitro and in vivo study. J Am Coll Cardiol.

[B12] Porter TR, LeVeen RF, Fox R, Kricsfeld A, Xie F (1996). Thrombolytic enhancement with perfluorocarbon-exposed sonicated dextrose albumin microbubbles. Am Heart J.

[B13] Mizushige K, Kondo I, Ohmori K, Hirao K, Matsuo H (1999). Enhancement of ultrasound-accelerated thrombolysis by echo contrast agents: dependence on microbubble structure. Ultrasound Med Biol.

[B14] Birnbaum Y, Luo H, Nagai T, Fishbein MC, Peterson TM, Li S, Kricsfeld D, Porter TR, Siegel RJ (1998). Noninvasive in vivo clot dissolution without a thrombolytic drug: recanalization of thrombosed iliofemoral arteries by transcutaneous ultrasound combined with intravenous infusion of microbubbles. Circulation.

[B15] Siegel RJ, Atar S, Fishbein MC, Brasch AV, Peterson TM, Nagai T, Pal D, Nishioka T, Chae JS, Birnbaum Y, Zanelli C, Luo H (2001). Noninvasive transcutaneous low frequency ultrasound enhances thrombolysis in peripheral and coronary arteries. Echocardiography.

[B16] Culp WC, Porter TR, Lowery J, Xie F, Roberson PK, Marky L (2004). Intracranial clot lysis with intravenous microbubbles and transcranial ultrasound in swine. Stroke.

[B17] Xie F, Tsutsui JM, Lof J, Unger EC, Johanning J, Culp WC, Matsunaga T, Porter TR (2005). Effectiveness of lipid microbubbles and ultrasound in declotting thrombosis. Ultrasound Med Biol.

[B18] Alexandrov AV, Molina CA, Grotta JC, Garami Z, Ford SR, Alvarez-Sabin J, Montaner J, Saqqur M, Demchuk AM, Moye LA, Hill MD, Wojner AW (2004). Ultrasound-enhanced systemic thrombolysis for acute ischemic stroke. N Engl J Med.

[B19] Molina CA, Ribo M, Rubiera M, Montaner J, Santamarina E, Delgado-Mederos R, Arenillas JF, Huertas R, Purroy F, Delgado P, Alvarez-Sabin J (2006). Microbubble administration accelerates clot lysis during continuous 2-MHz ultrasound monitoring in stroke patients treated with intravenous tissue plasminogen activator. Stroke.

[B20] Cohen MG, Tuero E, Bluguermann J, Kevorkian R, Berrocal DH, Carlevaro O, Picabea E, Hudson MP, Siegel RJ, Douthat L, Greenbaum AB, Echt D, Weaver WD, Grinfeld LR (2003). Transcutaneous ultrasound-facilitated coronary thrombolysis during acute myocardial infarction. Am J Cardiol.

[B21] De NVVC Richtlijnen Acute Coronaire Syndromen. http://www.nvvc.nl/UserFiles/File/Pdf/2001_acs.pdf.

[B22] Lichtveld RA, Hartman JAM, De Vries GMJ, Ten Wolde WLM (2005). Landelijk Protocol Ambulancezorg 2005.

[B23] EPAR for Luminity. http://www.emea.europa.eu/humandocs/PDFs/EPAR/luminity/065406en6.pdf.

[B24] 't Hof AW, Liem A, Suryapranata H, Hoorntje JC, de Boer MJ, Zijlstra F (1998). Angiographic assessment of myocardial reperfusion in patients treated with primary angioplasty for acute myocardial infarction: myocardial blush grade. Zwolle Myocardial Infarction Study Group. Circulation.

[B25] Gibson CM, Cannon CP, Daley WL, Dodge JT, Alexander B, Marble SJ, McCabe CH, Raymond L, Fortin T, Poole WK, Braunwald E (1996). TIMI frame count: a quantitative method of assessing coronary artery flow. Circulation.

[B26] Ross AM, Coyne KS, Reiner JS, Greenhouse SW, Fink C, Frey A, Moreyra E, Traboulsi M, Racine N, Riba AL, Thompson MA, Rohrbeck S, Lundergan CF (1999). A randomized trial comparing primary angioplasty with a strategy of short-acting thrombolysis and immediate planned rescue angioplasty in acute myocardial infarction: the PACT trial. PACT investigators. Plasminogen-activator Angioplasty Compatibility Trial. J Am Coll Cardiol.

